# Complement susceptibility in glutamine deprived breast cancer cells

**DOI:** 10.1186/1747-1028-2-20

**Published:** 2007-07-11

**Authors:** Bradley S Ellison, Mary KB Zanin, Robert J Boackle

**Affiliations:** 1 Department of Orthopaedic Surgery, The Ohio State University, 410W 10th Ave, Columbus Ohio 43210, USA; 2 Department of Biology, The Citadel, 171 Moultrie Street, Charleston, SC 29409, USA; 3 Department of Stomatology and Department of Immunology, Medical University of South Carolina, 171 Ashley Ave., Charleston, South Carolina 29425, USA

## Abstract

**Background:**

Membrane complement regulatory proteins (mCRPs) inhibit complement-mediated killing of human cells by human complement, a property that confers protection from complement to malignant breast cancer cells and that thwarts some immunotherapies. Metabolic mechanisms may come into play in protecting cancer cells from the complement system subsequent to relatively low levels of complement deposition.

**Results:**

In differentiating these mechanisms, two types of human breast cancer cell lines, MCF7 (adenocarcinoma) and Bcap37 (medullary carcinoma) were cell-cycle synchronized using glutamine-deprivation followed by restoration. These cells were examined for the expression of two mCRPs (CD59 and CD55), and for subsequent susceptibility to antibody-mediated complement-induced membrane damage. After glutamine restoration, MCF7 and Bcap37 cells were synchronized into the G2/M phase and an average increased expression of CD59 and CD55 occurred with a corresponding resistance to complement-mediated damage. Blocking CD59 inhibitory function with monoclonal antibody revealed that CD59 played a key role in protecting unsynchronized Bcap37 and MCF7 cancer cells from the complement membrane attack complex. Interestingly, glutamine-deprivation did not significantly affect the expression of proteins e.g., the surface level of CD59 or CD55, but did increase the susceptibility to complement-mediated killing. One possible explanation is that glutamine-deprivation may have slowed the turnover rate of mCRPs, preventing the cells from replacing pre-existing mCRPs, as they became neutralized by covalent C4b and C3b depositions.

**Conclusion:**

Taken together the findings are consistent with the conclusion that future immunotherapies should aim to achieve a highly specific and profound activation and deposition of complement as well as to disrupt the synthesis and expression of CD59 and CD55 by the cancer cells.

## Background

The complement system is a critical arm of the immune system that allows the system to eliminate pathogens. Complement is classically activated by the presence of antibodies on cell surface antigens. If allowed to proceed uninhibited, a complement activation cascade occurs that culminates in elimination of an antibody-coated cell via phagocytosis or lysis. Complement control mechanisms maximize the efficiency of the complement system in specifically targeting sensitized pathogens, while sparing incidental damage to nearby host cells. Indeed, membrane-associated complement regulatory proteins (mCRPs) are often over-expressed on host cells in areas of inflammation and restrict complement-mediated membrane damage after the inadvertent deposition of the complement components C4b or C3b [1]. In addition, malignant cancer cells express mCRPs [2,3] and may release soluble forms of selected mCRPs [4] which protect them against complement-mediated cell killing.

Fluid phase complement regulators, such as C1-inhibitor prevent the unnecessary consumption and depletion of soluble complement components, so as to allow a more effective and specifically directed complement-mediated attack on sensitized pathogens. However in the absence of sufficient levels of antibody deposition, this and other complement control mechanisms tend to restrict the ability of complement to eliminate cancer cells [2,5-9]. Current therapeutic mAbs as well as endogenous low affinity IgG antibodies to cancer cells often recruit the complement component C1qr_2_s_2 _with such low avidity that serum C1-inhibitor is able to rapidly inhibit activated C1r and C1s, and in most cases quickly remove the entire C1qr_2_s_2 _complex from the antibody-coated cell surface, resulting in only a trace level of C4b-deposition [10-12]. Meanwhile, CD55 and CD46 on malignant cells restrict deposited C4b and C3b, and CD59 inhibits complement membrane attack complex formation, therein protecting cancer cells from membrane damage [13,14]. When the complement cascade is strongly activated, the complement components C4b and C3b bind to mCRPs and inactivate them, but low levels of complement depositions are incapable of neutralizing sufficient percentages of the expressed mCRPs on the surface of the cancer cells. Indeed, repeated low level, albeit ineffective, complement-depositions are capable of inducing metabolic consequences that may result in an undesirable increase in resistance to apoptotic influences [15,16] and could enhance resistance to complement-mediated killing [17,18], therein providing an acquired advantage for the surviving cancer cell populations [19]. Therefore, when designing antibody therapies to utilize complement in the elimination of malignant cancer cells, highly effective classical pathway activation may be needed to mediate sufficient C4b and C3b depositions to covalently bind and block the function of mCRPs on the targeted cancer cell surface.

Cell cycle dependent phenomena and resultant expression of targeted antigens have been associated with variant susceptibilities to complement-mediated lysis [20-22]. However, many of these studies were conducted just as CD59 and CD55 were being fully characterized and several studies used cross-species complement. In addition, few reports have examined the effect of cell-cycle synchronization on the expression of mCRPs in breast cancer cell lines. In this study, the expression levels of CD59 and CD55 were measured in two different types of human breast cancer cell lines, a human breast adenocarcinoma cell line MCF7 [23] and a human breast medullary carcinoma cell line Bcap37 [24] before and after cell cycle synchronization using glutamine-deprivation and restoration. The expression levels of CD59 and CD55 were correlated to susceptibility to human complement-mediated lysis following complement activation. Complement was activated by exposing cancer cells to excess levels of polyclonal rabbit antibodies to β_2_-microglobulin, a stably and abundantly expressed antigen on cancer cell line surfaces [23,25]. Use of this polyclonal antibody to activate C1 blocks the rapid entrance and action of C1-inhibitor [10,11] allowing sufficient progression of the complement cascade so as to allow a relative measurement of the impact of mCRPs on complement-mediated cell damage.

In these studies we found that glutamine deprivation increases breast cancer cell susceptibility to complement-mediated killing. We also found that subsequent glutamine restoration produces cancer cells that substantially up-regulate the expression of the mCRPs CD59 and CD55 on the cell surface and that these glutamine-restored cancer cells are even more resistant to complement-mediated damage than unsynchronized breast cancer cells.

## Results

### Complement-mediated lysis of antibody-coated breast cancer cells as a function of the level of normal human serum as a source of complement

Normal human serum (NHS) is a readily available and reliable source that contains the entire battery of complement proteins needed to evoke the immunological cascade of antibody-directed complement-mediated cell lysis. As lytic damage to the cell membrane occurs, intracellular enzymes and elements are released into the extra-cellular domain, allowing them to become markers in determining the extent of cellular damage incurred during complement-mediated lysis. In this manner, a commercially available Lactate Dehydrogenase (LDH) assay (Sigma Diagnostics) was employed to detect the extent of total complement-mediated cell lysis.

**Figure 1:**
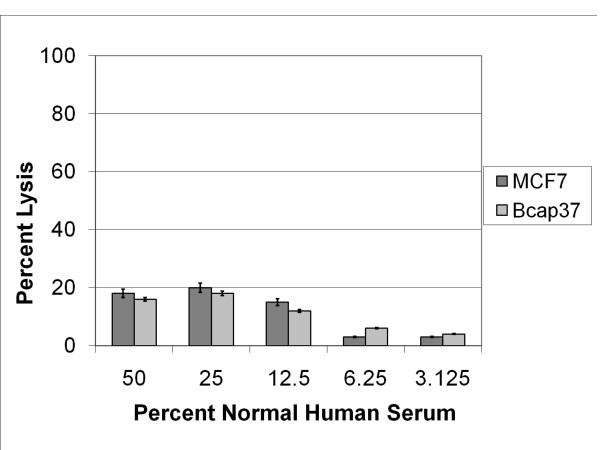
Optimal NHS Dilution for Complement-mediated lysis of human breast cancer cell lines. The Bcap37 and MCF7 cells (1 × 10^5^) were sensitized 20 μg rabbit antibody to β_2_-microglobulin and incubated with specified dilutions of normal human serum (NHS). After a total 4.5 hours of incubation at 37°C, supernatants were collected and tested for LDH activity to determine percent lysis.

In NHS, however, various levels of background LDH activity can be detected. Serial dilutions of NHS were developed to identify the most appropriate concentration of NHS that could be used in further experimentation demonstrating minimal background LDH activity, while optimizing detection of LDH activity as an index of antibody-directed complement-mediated lysis. II BothThe use of 25% NHS as a source of complement provided the maximal level of complement-mediated lysis [26], while allowing an acceptable serum-LDH background. Detection of complement-mediated membrane damage to anti-β_2_-microglobulin sensitized cancer cells, monitored via release of LDH (and Trypan Blue exclusion), indicated that 20% of the MCF7 cells and 18% of the Bcap37 cells were lysed (Figure 1). As NHS was further diluted, both MCF7 and Bcap37 cells became increasingly resistant to complement-mediated membrane damage. Therefore 25% NHS as a source of complement was used throughout the remaining studies.

### Neutralization of CD59 enhanced complement-mediated lysis of breast cancer cells

**Figure 2:**
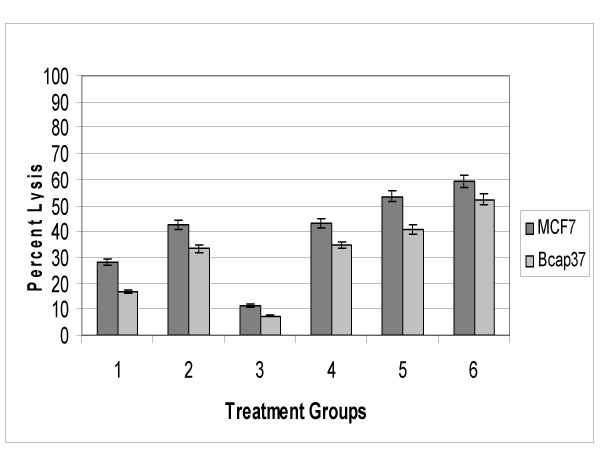
Neutralization of CD59 enhanced complement-mediated lysis. In A through D, Bcap37 and MCF7 cells (1 × 10^5^) were sensitized with 20 μg of rabbit antibody to β_2_-microglobulin (anti-β2M) with or without specified doses of mAb-YTH53.1 (anti-CD59). Background LDH activity was obtained with unsensitized cells similarly incubated with 25% NHS for 4.5 hours to provide a numerical value that was subtracted from experimental values. Experimental antibody combinations are represented as follows: 1) 10 μg anti-β2M alone (28.0% lysis ± 1.7). 2) 20 μg anti-β2M alone (42.3% lysis ± 2.9). 3) 20 μg mAb-YTH53.1 alone (11.3% lysis ± 1.5). 4) 20 μg anti-β2M + 5 μg mAb-YTH53.1 (43.3% lysis ± 2.1). 5) 20 μg anti-β2M + 10 μg mAb-YTH53.1 (53% lysis ± 4.8). 6) 20 μg anti-β2M + 20 μg mAb-YTH53.1 (59% lysis ± 4.9).

The cell surface marker CD59 (Protectin) prevents formation of the membrane attack complex, the terminal arm of the activated complement system responsible for inducing cell lysis. Thus, CD59 is considered one of the most important innate defense mechanisms cells have against complement activation. The YTH53.1 monoclonal antibody (Serotec, Raleigh, NC) has been proven to bind to CD59 on the surface of human cells and neutralize the complement inhibitory function of CD59 (37,38). Our objective was to determine the specific degree of protection CD59 offers MCF7 and Bcap37 breast cancer cells from antibody-directed complement-mediated damage. The MCF7 and Bcap37 cells received the combined treatment of YTH53.1 along with anti-β_2_-microglobulin antibodies followed by 25% NHS. Cells treated with only the YTH53.1 anti-CD59 monoclonal antibody demonstrated only 7% lysis for Bcap37 and 11% lysis for MCF7 cells (Figure 2, Treatment group 3). The relatively small levels of lysis attributed to YTH53.1 alone were due to inhibition of CD59 function and mild complement activation and regarded as background lysis. When treated with only 20 μg of rabbit polyclonal anti-β_2_-microglobulin antibodies, the percent lysis for Bcap37 cells was 33% and for MCF7 cells was 42%. However, when 20 μg of YTH53.1 anti-CD59 monoclonal antibodies were combined with the anti-β_2_-microglobulin antibodies, the percent lysis significantly increased to 52% for Bcap37 cells (Figure 2, Treatment groups B and F: p value = 0.009) and 59% for MCF7 cells (Figure 2, Treatment groups B and F: p value = 0.005). Thus, neutralization of CD59 function with YTH53.1 significantly increased complement-mediated lysis of breast cancer cells.

No significant difference was observed between Bcap37 and MCF7 cells treated with 20 μg of YTH53.1 and either 10 or 20 μg of rabbit polyclonal anti-β_2_-microglobulin antibodies. However, Bcap37 cells treated with anti-β_2_-microglobulin antibodies and 5 μg of YTH53.1 demonstrated 34% lysis, while cells treated with anti-β_2_-microglobulin antibodies and 20 μg of YTH53.1 demonstrated 52% lysis (Figure 2, Treatment groups D and F; p value = 0.05). Likewise, MCF7 cells treated with anti-β_2_-microglobulin antibodies and 5 μg of YTH53.1 demonstrated 43% lysis, while cells treated with anti-β_2_-microglobulin antibodies and 20 μg of YTH53.1 demonstrated 59% lysis (Figure 2, Treatment groups D and F; p value = 0.02). With the YTH53.1 antibody demonstrating a dose-response, the 20 μg of YTH53.1 was selected as an effective amount in eliciting complement-mediated lysis for further experimentation.

### Cell cycle synchronization with glutamine deprivation and restoration

Using flow cytometry, cells in different phases of the cell cycle are identified based on the amount of DNA present inside the cell, since duplication of genetic material occurs during progression of the cell cycle when a diploid cell prepares for mitotic division. Specifically, propidium iodide intercalates within DNA providing a fluorescent marker that allows measurement of total DNA material by flow cytometry. Diploid cells in the G0-G1 phase are denoted as having a total DNA content of 2C, while cells in S phase have a total DNA content between 2C and 4C, and cells in G2-M phase have a total DNA content of 4C. Discrimination between G0 and G1 phases is not possible because the amount of DNA is identical (2C) for cells in either the G0 or G1 phase, thus cells with 2C DNA were grouped together as G0-G1 phase. Similarly, the amount of DNA is identical for cells in either the G2 or M phase, thus cells with 4C DNA were grouped together as G2-M phase.

**Figure 3:**
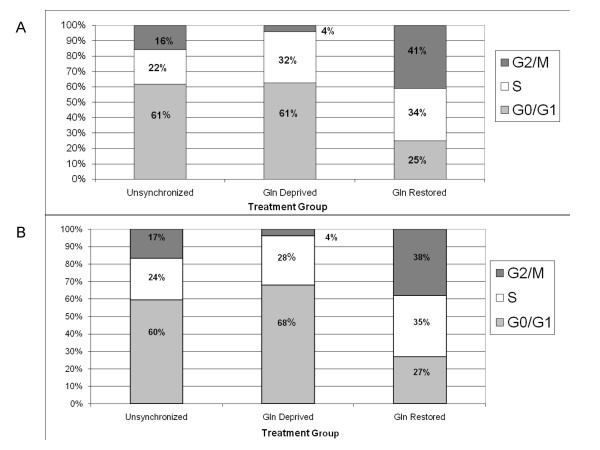
Cell cycle distribution for breast cancer cells subjected to glutamine-mediated synchronization. Data are depicted as representative means for three independent experiments. Bcap37 cells (Figure 3a) and MCF7 cells (Figure 3b) were grown to confluency and either maintained in media with glutamine (Unsynchronized) or without glutamine for 48 hours (Gln Deprived), or without glutamine for 48 hours but then followed by 8 hours with glutamine (Gln Restored). All cells were harvested and treated with 0.5 mg/ml propidium iodide to determine cell cycle distribution by FACS analyses.

Glutamine is an amino acid routinely added to cell culture media due to its importance in the regulation of cell metabolism through its association with the synthesis of both protein and DNA (27). Glutamine-deprivation has been used as a method of arresting cells in the G0 phase of the cell cycle (39). When glutamine is restored to the culture media, the cells are able to then proceed unimpeded through the cell cycle. After Bcap37 and MCF7 cells had been deprived of glutamine for 48 hours, 2 mM L-glutamine was added to the culture medium. Distinct cell cycle profiles were characterized for Bcap37 and MCF7 cells subjected to glutamine synchronization when compared with unsynchronized cells. Distribution of population averages were calculated for G0-G1 phase, S phase, and G2-M phase among unsynchronized and synchronized cells. Unsynchronized Bcap37 cells demonstrated 61% in G0-G1 phase, 22% in S phase and 16% in G2-M phase (figure 3a). Glutamine deprived Bcap37 cells demonstrated 61% in G0-G1 phase, 32% in S phase and 4% in G2-M phase. Glutamine restored Bcap37 cells demonstrated 25% in G0-G1 phase, 34% in S phase and 41% in G2-M phase. Unsynchronized MCF7 cells demonstrated 60% in G0-G1 phase, 24% in S phase and 17% in G2-M phase (figure 3a). Glutamine deprived MCF7 cells demonstrated 68% in G0-G1 phase, 24% in S phase and 4% in G2-M phase. Glutamine restored MCF7 cells demonstrated 27% in G0-G1 phase, 35% in S phase and 38% in G2-M phase. Thus, the cell cycle profile changes significantly depending on the presence or absence of glutamine revealing that glutamine depletion synchronizes cells by arresting progression predominantly in G0-G1 phase. However, upon restoration of glutamine the cell cycle profile is further altered with an abrupt forward progression into G2-M phase. Therefore, glutamine depletion and restoration affords the unique ability to synchronize the cell cycle, resulting in significant shifts in the proportion of MCF7 and Bcap37 cells in G0-G1 phase, S phase or G2-M phase. Further, cells receiving glutamine following glutamine deprivation demonstrated a common shift in the proportion of cells in the quiescent G0-G1 phase to active mitotic proliferation of G2-M phase, the most characteristic stage of neoplastic transformation.

### The effect of glutamine deprivation and restoration on the expression of CD59 and CD55

**Figure 4:**
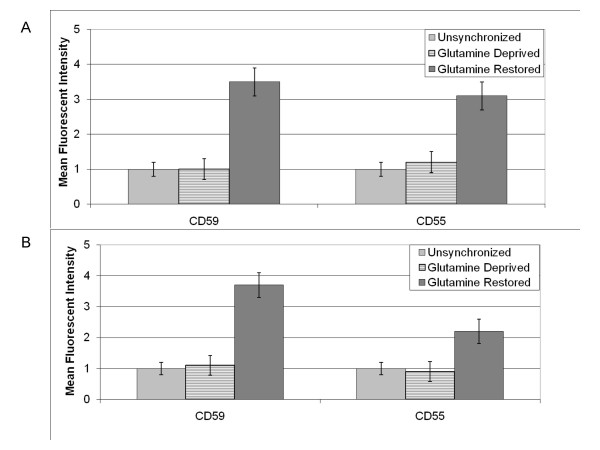
Expression of CD59 and CD55 in breast cancer cells subjected to glutamine-mediated synchronization. Data is depicted as representative means for three independent experiments. Bcap37 cells (Figure 4a) and MCF7 cells (Figure 4b) were grown to confluency and either maintained with glutamine (Unsynchronized) or in the same media without glutamine for 48 hours (Glutamine Deprived), or in the same media without glutamine for 48 hours followed by 8 hours with glutamine (Glutamine Restored). In all experimental groups for Bcap37 and MCF7 cells, CD59 was quantified using FITC-conjugated anti-CD59 mouse monoclonal antibody and CD55 was quantified using FITC-conjugated anti-CD55 mouse monoclonal antibody.

With glutamine synchronization resulting in significant shifts in the cell cycle profiles for Bcap37 and MCF7 cells, the cells were then examined to determine if glutamine synchronization impacted CD59 and CD55 expression. Bcap37 and MCF7 cells were synchronized with glutamine deprivation for 48 hours, then subsequently received 2 mM L-glutamine for 8 hours. Following glutamine restoration, cells were harvested and prepared analysis by flow cytometry. Using the ModFit LT software, the Mean Fluorescence Intensity (MFI) was examined with the cell acquisition being approximately 5,000 cells for each treatment group (data not shown). The MFI was examined for CD59, CD55 and the appropriate negative isotype controls for unsynchronized and glutamine restored cells. The negative background isotype controls (*non-specific FITC-labeled mouse immunoglobulins, IgG2a and IgG*) revealed negligible cell surface binding. Compared with unsynchronized cells, glutamine restoration resulted in a 3.5-fold increase in and a 3.1-fold increase in CD55 expression for Bcap37 cells (Figure 4a). Similar results were observed for MCF7 cells upon glutamine-restorationwith a 3.7-fold increase in CD59 expression and a 2.2-fold increase in CD55 expression (Figure 4b). Thus, glutamine restoration resulted in increased CD59 and CD55 expression for Bcap37 and MCF7 cells when controlled for cell number. Therefore, glutamine restoration not only induced Bcap37 and MCF7 cells to rapidly progress through the cell cycle, but also augmented the levels of CD59 and CD55 expression along with the mitotic index.

### Comparison of CD59 and CD55 expression in G0-G1 and G2-M phases of the cell cycle

**Figure 5:**
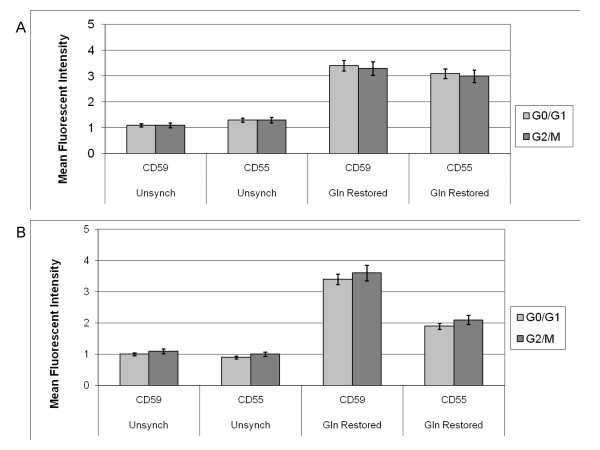
CD59 and CD55 expression in G0-G1 and G2-M subpopulations. Data is depicted as representative means for three independent experiments. Unsynchronized Bcap37 cells (Figure 5a) and MCF7 cells (Figure 5b) were grown to confluency and maintained in media supplemented with 2 mM L-glutamine (Unsynch). Synchronized cells were grown to confluency in normal media and then maintained in media without glutamine for 48 hours, followed by 8 hours in media supplemented with 2 mM L-glutamine (Gln Restored). Cells were then harvested, treated with propidium iodide, incubated with either FITC-conjugated antibodies to CD59 or to CD55 and prepared for flow cytometric sorting and analysis.

Given the distinct cell cycle profiles observed with glutamine-deprived and glutamine-restored cells, the possibility existed that the increase in CD59 and CD55 expression might be directly related to the cell cycle phase. To determine if CD59 or CD55 expression was phase specific, flow cytometry was used to isolate the G0-G1 and the G2-M subpopulations of Bcap37 and MCF7 cells within each synchronization group. During Cell Analysis using the FACSCalibur device and ModFit LT software, the distinct populations of cells were identified in either G0-G1 or G2-M based on channel values dictated by the amount of propidium iodide intercalated within DNA. All diploid cells (2C) were identified, then separated according to channel values based on propidium iodide fluorescence at 585 nm (data not shown). Cells identified as G0-G1 had channel values ranging approximately from 25 to 75. Cells identified as G2-M had channel values ranging approximately from 140 to 175. Gating was performed manually after identification of G0-1 and G2-M populations to further determine CD59 and CD55 MFI among these distinct populations of cells at different stages in the cell cycle. Using the ModFit LT software, the Mean Fluorescence Intensity (MFI) was examined with the cell acquisition being approximately 5,000 cells for each treatment group (data not shown). The MFI was examined for CD59, CD55 and the appropriate negative isotype controls in the G0-G1 and G2-M channel ranges for unsynchronized and glutamine restored cells. The negative background isotype controls (*non-specific FITC-labeled mouse immunoglobulins, IgG2a and IgG*) revealed negligible cell surface binding. Since glutamine-deprived Bcap37 and MCF7 cells had only 4% of total cells in G2-M, an inadequate number of cells were available for determination of MFI for CD59 and CD55. However, the G2-M subpopulations for the unsynchronized and glutamine restored Bcap37 and MCF7 cells contained a sufficient number of cells for analysis. When comparing the MFI for CD59 and CD55 between G0-G1 and G2-M subpopulations of cells in different phases of the cell cycle, no significant difference in mCRP expression was observed for Bcap37 or MCF7 cells (Figures 5a and 5b). Therefore, the increased expression of CD59 and CD55 observed with glutamine restoration appears to not be directly related to the increased proportion of cells in the G2-M phase of the cell cycle. Rather, other factors that are not cell cycle-dependent, yet associated with the metabolic proliferation stimulated by glutamine restoration are responsible for the increased expression of mCRPs.

### Influence of glutamine deprivation or restoration on complement susceptibility

**Figure 6:**
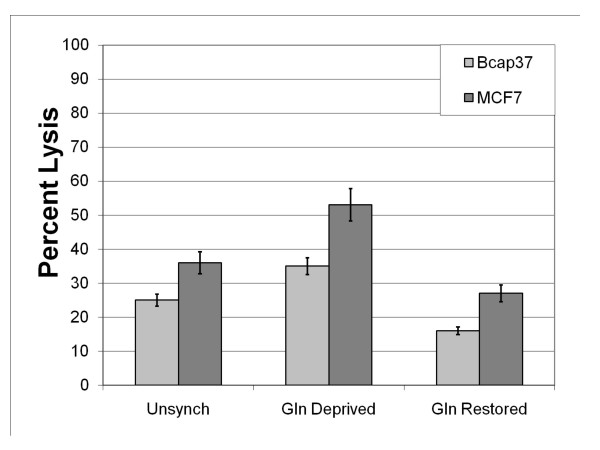
The effect of glutamine-mediated synchronization on complement susceptibility in breast cancer cell lines. All populations of cells were grown to confluency in separate culture flasks. Unsynchronized cells were maintained in 10% FBS DMEM supplemented with 2 mM L-glutamine, while some cells were subsequently maintained in glutamine deficient-media for 48 hours (Gln Deprived), and of this group, some were subsequently supplemented with 2 mM L-glutamine for 8 hours (Gln Restored). All populations of cells were sensitized with 20 μg of rabbit polyclonal antibody to β_2_-microglobulin and subjected to 25% fresh normal human serum (NHS). After 4.5 hours of incubation with NHS at 37°C, supernatants were collected and tested for LDH activity to determine percent lysis.

With the presence or absence of glutamine impacting the expression levels of mCRPs, the susceptibility of breast cancer cells to complement-mediated lysis also is likely dependent on the relative level of glutamine in the cellular microenvironment. To determine if changes in expression levels of CD59 and CD55 altered their susceptibility to complement-mediated lysis, unsynchronized, glutamine-deprived and glutamine-restored cells were treated with complement-activating antibodies and incubated with NHS for 4 hours to determine the percentage of complement-mediated lysis. When treated with 20 μg of rabbit polyclonal anti-β_2_-microglobulin antibodies, unsynchronized Bcap37 cells demonstrated 26% lysis (Figure 6). However, when Bcap37 cells were cultured in a glutamine-deficient medium, complement-mediated damage significantly increased to 37% (p value = 0.007). Further, when glutamine was restored to the media following a 48-hour period of deprivation, Bcap37 cells demonstrated 17% lysis, a significant reduction in complement susceptibility compared to either unsynchronized or glutamine deprived cells (p values = 0.05 and 0.003, respectively). The impact of glutamine synchronization affected the MCF7 cells similarly. When treated with 20 μg of rabbit polyclonal anti-β_2_-microglobulin antibodies, unsynchronized MCF7 cells demonstrated 37% lysis. Yet, MCF7 cells cultured in glutamine-deficient media demonstrated a significant increase in complement susceptibility with 54% lysis (p value = 0.01). Additionally, when glutamine is restored to the media, MCF7 cells demonstrated a significant decrease in complement susceptibility compared with glutamine-deprived cells illustrated by 26% lysis (p value = 0.007).

As expected, glutamine restored Bcap37 and MCF7 cells with increased CD59 and CD55 expression exhibited heightened resistance to complement-mediated lysis. Interestingly, when compared with unsynchronized cells, glutamine deprived Bcap37 and MCF7 cells demonstrated increased susceptibility to complement-mediated lysis although expression levels of CD59 and CD55 were similar (figures 4a and 4b). Thus, the increased expression of CD59 and CD55 enhance complement resistance, however, other factors associated with glutamine deprivation beyond expression levels of mCRPs also influence complement susceptibility.

### Neutralization of CD59 augments complement-mediated lysis following glutamine synchronization

Glutamine synchronization altered the expression levels of mCRPs, cell cycle profiles, and complement susceptibility among breast cancer cells, suggesting microenvironmental perturbations likely influence complement-activating antibody-directed breast cancer therapies.

One of the most effective mCRPs is CD59, which functions to prevent formation of the membrane attack complex specifically blocking the terminal arm of the complement cascade. The YTH53.1 monoclonal antibody (Serotec, Raleigh, NC) has been proven to bind to CD59 on the surface of human cells and neutralize its complement inhibitory function (37,38). Our objective was to determine the specific degree of protection CD59 offers Bcap37 and MCF7 breast cancer cells from antibody-directed complement-mediated damage when simultaneously challenged with glutamine synchronization.

**Figure 7:**
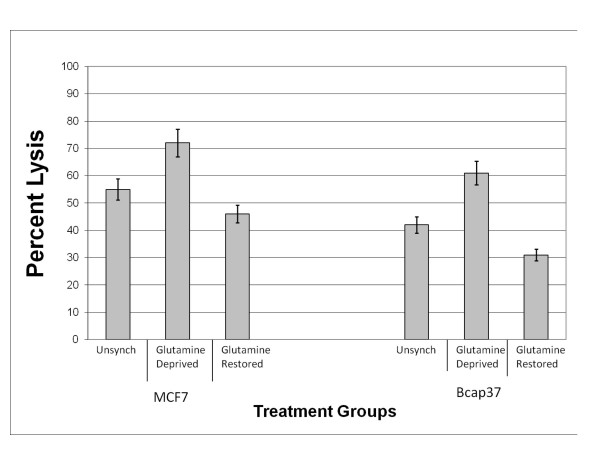
Percent Lysis following Glutamine Synchronization and Neutralization of CD59. Treatment groups were prepared as outlined for unsynchronized, glutamine deprived and glutamine restored populations of Bcap37 and MCF7 cells. Cells were sensitized with 20 μg of rabbit polyclonal antibody to β_2_-microglobulin and subjected to 25% fresh normal human serum (NHS) in combination with 20 μg of YTH53.1 rat monoclonal antibody to CD59. After 4 hours of incubation with NHS at 37°C, supernatants were collected and tested for LDH activity to determine percent lysis. Please note the increase in percent lysis for both cell lines as compared to Figure 6.

For Bcap37 and MCF7 cells, the YTH53.1 monoclonal antibody was used in combination with complement activating anti-β_2_-microglobulin antibodies and 25% NHS. Unsynchronized Bcap37 cells demonstrated 42% lysis (Figure 7). Following glutamine deprivation, Bcap37 cells revealed a significant increase in complement-mediated lysis of 61% (p value = 0.05). Additionally, glutamine restoration resulted in 31% lysis, revealing decreased complement susceptibility when compared with the unsynchronized and glutamine-deprived treatment groups (p values = 0.02 and 0.01, respectively). (All of these lysis values for Bcap37 cells are higher than those of cells treated with only anti-β_2_-microglobulin as seen in Figure 6, where unsynchronized Bcap37 cells demonstrated 26% lysis, glutamine deprived Bcap37 cells demonstrated 37% lysis, and glutamine restored Bcap37 cells demonstrated 17% lysis). Similar observations were seen with MCF7 cells, with unsynchronized cells demonstrating 55% lysis. Following glutamine deprivation, MCF7 cells showed a significant increase in complement-mediated lysis of 72% (p value = 0.001). Further, glutamine restoration resulted in decreased complement susceptibility with 46% lysis, a significant decrease compared with unsynchronized and glutamine-deprived MCF7 cells (p value = 0.02 and 0.01, respectively). (All of these lysis values for MCF7 cells are higher than those of cells treated with only anti-β_2_-microglobulin as seen in Figure 6, where unsynchronized MCF7 cells demonstrated 37% lysis, glutamine deprived MCF7 cells demonstrated 54% lysis, and glutamine restored MCF7 cells demonstrated 26% lysis).

In view of these results, CD59 offers significant resistance to complement-mediated lysis regardless of whether cells are subjected to glutamine synchronization or not. However, when Bcap37 and MCF7 cells are faced with potent complement activation in the face of glutamine deprivation and CD59 neutralization, complement susceptibility is markedly increased. Conversely, restoration of glutamine seemingly reverses the increased complement susceptibility observed with glutamine deprivation. However, neutralization of CD59 combined with potent complement activation still elicited appreciable complement-mediated lysis when compared to glutamine-restored cells not treated with YTH53.1 (Figure 6). Additionally, glutamine-restored Bcap37 and MCF7 cells exhibited increased complement resistance than their unsynchronized counterparts, even when CD59 was neutralized for both treatment groups. Therefore, glutamine restoration not only increases complement resistance by elevating CD59 expression, but also likely induced other yet unidentified intracellular responses that provided additional resistance to complement-mediated lysis.

## Discussion

Membrane complement regulatory proteins (mCRPs) that are expressed on breast cancer cells, as well as normal cells provide exquisite protection from complement-mediated lysis [13,28-30], after inadvertent C4b or C3b deposition or after a marginal initiation of the classical pathway subsequent to sensitization of cancer cells by exogenous monoclonal antibodies or low affinity endogenous antibodies. Should the complement cascade override the effects of CD55 (and CD46, another mCRP) and manage to activate the terminal complement components on cancer cell membranes, functional CD59 molecules bind to human C8 and C9, during the attempt to properly assemble the membrane attack complex [31]. By preventing complement-mediated membrane damage, CD59 conveys an immunological privilege to both normal host and neoplastic cells [1,5,6,32]. The soluble complement inhibitor, C1-inhibitor, acts at the sensitized surface to remove C1 and block the classical pathway, but a highly effective initiation of the classical complement pathway by abundant levels of complement-activating polyclonal antibodies to numerous proximate antigenic determinants on cancer cell surfaces may override C1-inhibitor [10-12], and, as shown here, may generate a profound fixation of complement that results in a significant percentage of cell killing as measured by the release of LDH and uptake of Trypan Blue.

In these experiments, serum is the source of complement, but the serum also contains C1-inhibitor. The dilution of serum from 50% to 25% caused an increased complement-mediated lysis of the sensitized cancer cells (Figure 1). We have reported previously that C1-inhibitor function is the rate-limiting step in the initiation of the classical pathway [10,11]. Therefore we suggest that under the conditions of our experiment, the level of functional C1-inhibitor was reduced to a defined point that allowed an increase in C1 deposition, yet did not significantly compromise C1-inhibitor's ability to control fluid-phase C1 activation, which would otherwise result in depletion of soluble complement components.

It may seem unlikely that cells expressing significant levels of mCRPs should suffer any complement-mediated lysis. A reasonable speculation is that the direct covalent deposition of complement components (e.g., C4b and C3b) onto a percentage of the mCRPs can inhibit the functions of those mCRPs, therein creating pivotal conditions that enable complement-mediated membrane damage. High dilutions of serum also dilute complement components (and complement control proteins) resulting in a progressively lower level of complement deposition, which could be more easily controlled by the functional mCRPs on the cancer cell surface. For these reasons we chose to apply excess levels of rabbit polyclonal antibodies to β_2_-microglobulin, a stable and ubiquitously expressed cell surface marker. We used 25% fresh human serum in our study of the complement-mediated killing of two different types of breast cancer cell lines, a breast adenocarcinoma cell line MCF7 [23], and a human breast medullary carcinoma cell line, Bcap37. These conditions allowed an assessment of the impact of glutamine-deprivation on cell cycle kinetics, mCRP expression and complement susceptibility in a breast cancer model.

Breast cancer cell lines subjected to a 48-hour period of glutamine-deprivation were unable to progress through mitosis, with the majority of cells trapped in G0-G1 phase. Trypan Blue exclusion verified the viability of all cells at harvesting following control and experimental treatments, including glutamine-deprivation. Restoration of glutamine to glutamine-deprived cells induced a substantial surge in cell cycle progression, with an increased proportion of cells in the G2-M phase. However, our analysis reveals that the expression of CD59 and CD55 is not dependent upon phase of the cell cycle, with G0-G1 phase cells demonstrating no significant difference in mCRP expression compared with G2-M phase cells. Yet, the restoration of glutamine to glutamine-deprived cells stimulated increased mCRP expression suggesting that CD59 and CD55 expression may be related more closely to metabolic proliferation rather than dependent on specific phases of the cell cycle. Coincidently, the rapidly proliferating glutamine-restored breast cancer cells demonstrated increased complement resistance in association with the increased expression of CD59 and CD55. When accounting for overall cell number, the relative surface expression level of CD59 and CD55 was observed to increase appreciably in glutamine-restored breast cancer cells, which explains the associated increased complement resistance. However, other unidentified factors that impact complement susceptibility, such as membrane permeability and cytoskeleton integrity, could have also been influenced by glutamine restoration. Nonetheless, increased CD59 and CD55 expression protect breast cancer cells form antibody-directed complement activation. Particularly, the importance of CD59 was underscored in our experiments. With CD59 neutralized, unsynchronized Bcap37 and MCF7 cells demonstrated 42% and 55% complement lysis, respectively. However, with functional CD59 activity unsynchronized Bcap37 and MCF7 cells demonstrated 26% and 37% complement lysis, respectively, indicating CD59 contributes significantly to complement resistance in unsynchronized breast cancer cells. Other studies have suggested CD59 is one of the major factors in providing host cells protection from complement activation (29, 37, 38).

Interestingly, neutralization of CD59 in breast cancer cells deprived of glutamine resulted in maximal complement-mediated damage, with lysis of 61% of Bcap37 and 72% of MCF7 cells. The heightened complement susceptibility associated with glutamine deprivation was more than completely reversed with glutamine restoration, even if CD59 was neutralized or not. This suggests factors other than mCRPs are recruited that provide additional complement resistance to cells entering hypermetabolic states, similar to the phenomenon observed with glutamine restoration. Similar to our results, other studies have related metabolic deficiencies with increased complement susceptibility due to structurally compromised cell membranes [22,33,34], which could be more easily disrupted by antibody-directed complement activation. Further studies will be needed to examine and compare each of these possibilities under the metabolic conditions induced by glutamine-deprivation.

Chemotherapeutic agents preferentially target rapidly proliferating cancer cells with the intention of inducing a tumoricidal response while minimizing collateral host damage. Many chemotherapeutic agents alter the metabolic state and cytokinetic profile in a manner not unlike glutamine-deprivation [20,21,36]. In future studies, the specific impact chemotherapeutic agents impose on complement susceptibility when cells are challenged with antibody-directed complement activation could provide valuable information in designing more effective immunotherapeutic treatments. From our studies, a theoretical advantage may be obtained from combining medical agents which increase complement susceptibility of cancer cells, either through reduced mCRP expression or altered metabolic profiles, with complement activating antibody-directed immunotherapeutic treatments.

In terms of the immunological privilege often associated with neoplasms, low levels of sustained, albeit ineffective complement deposition (e.g., at the tumor site) have been shown to propagate cancer cells that are more resistant to apoptotic signals [19] and concomitantly the surviving cells become even more resistant to complement-mediated damage [17,18]. This could partially explain why limited non-lethal *in vivo *complement activation localized to the tumor site could exacerbate the inherent pathology associated with malignancy, therein-complicating treatment efforts [18]. Malignant cancer cells also liberate soluble forms of complement mCRPs that maintain their complement-restrictive activities [4]. Thus, the evasive properties of malignant cancer cells may be attributed to an interrelated, yet not fully understood, complex of resistance mechanisms that promote cell survival and immunological privilege. Future studies will hopefully delineate the impact of specific molecular pathways associated with non-lethal complement activation and glutamine deprivation or chemotherapy on the immunological privilege and chemotherapeutic resistance commonly identified in malignant breast cancer cells.

## Conclusion

Glutamine deprivation resulted in increased complement susceptibility and altered cell cycle profiles in breast cancer cells. However, glutamine restoration led to a rapid progression through the mitotic cell cycle, as well as increased CD59 and CD55 expression, which correlated with increased resistance to antibody-directed complement activation. When CD59 is neutralized, both glutamine-deprived and glutamine-restored breast cancer cells demonstrated increased complement susceptibility. These findings are important when considering the design of antibody-directed immunotherapies which attempt to selectively target cancer cells for complement-mediated killing.

## Methods

### Cell culture media and glutamine synchronization

The human breast adenocarcinoma cell line MCF7 [23] and the human breast medullary carcinoma cell line Bcap37 [24] were obtained from the Hollings Cancer Center at the Medical University of South Carolina. The MCF7 cells have been extensively examined for expression of β_2_-microglobulin [23]. Adenocarcinomas are much more common than medullary mammary carcinomas, however these two very distinct cell types were employed for comparative purposes. Cell lines were customarily maintained in Delbecco's Modified Eagle Medium (DMEM) supplemented with 10% heat-inactivated fetal bovine serum (FBS), 2 mM L-glutamine, 50 units/ml of penicillin and 50 μg/ml of streptomycin at 37°C in humidified air with 5% carbon dioxide. All media and supplements were obtained from Fischer Scientific.

Cells were grown to confluence and then to establish treatment groups, approximately 1 × 10^5 ^MCF7 or Bcap37 cells were inoculated onto 48-well plates. For 24 hours, treatment groups were allowed to adhere to the plate surface in DMEM media supplemented with 10% FBS, antibiotics and 1 mM L-glutamine at 37°C. Next, the media was removed and cells were washed with sterile PBS. As is well-known, L-glutamine is an essential component for cell growth (Minamoto, et al, Cytotechnology. 1991;5 Suppl 2:S35-51). Treatment groups were then established based on glutamine supplementation and identified as unsynchronized, glutamine deprived or glutamine restored. "Unsynchronized" cells continued to grow uninterrupted for 48 hours in standard DMEM culture media with additional supplements as detailed above. The "glutamine deprivation" group received DMEM with 5% FBS, antibiotics, but no glutamine for 48 hours. The conversion from 10% FBS to 5% FBS minimized untoward exposure of cells to extraneous L-glutamine occasionally contained in FBS preparations, but contains an adequate amount of the essential serum elements required to support basic cellular activities for 48 hours [39]. Additionally, we have observed Bcap37 and MCF7 cell lines can be continuously grown in DMEM supplemented with 5% FBS and 2 mM L-glutamine. The "glutamine restored" cells were treated identical to the "glutamine deprived" cells for 48 hours, but then received DMEM with 10% FBS and 2 mM L-glutamine for 8 hours before harvesting. The 48-hour period of glutamine-deprivation synchronized cells in a quiescent G0-G1 phase of the cell cycle, allowing comparison of glutamine-deprived cells with unsynchronized cells and cells treated with glutamine deprivation followed by glutamine restoration.

### Complement-activating antibodies and normal human serum

Approximately 1 × 10^5 ^MCF7 or Bcap37 cells were inoculated onto 48-well plates with each well supplemented with DMEM media with 10% fetal bovine serum (FBS), antibiotics, and glutamine. To induce complement activation, cells were sensitized with either 10 μg (20 μg/ml) or 20 μg (40 μg/ml) of rabbit polyclonal antibody to human β_2_-microglobulin (anti-β 2M, Accurate Inc.), a ubiquitously expressed cell surface marker associated with Major Histocompatibility Complex-1 antigen [25]. When indicated, antibody-mediated neutralization of CD59 function was achieved using YTH53.1 (Serotec), a rat monoclonal antibody to CD59 proven to neutralize its complement-inhibitory function (37,38). The YTH53.1 has demonstrated high specificity for human CD59 and effectively neutralizes it complement inhibition properties, thus negative antibody controls testing the specificity of YTH53.1 were not necessary (37,38, 42, 43). Specified amounts of 0, 5, 10, or 20 μg representing 0 μg/ml, 10 μg/ml, 20 μg/ml and 40 μg/ml absolute concentrations of YTH53.1 were added in combination with an optimized level (20 μg) of rabbit polyclonal anti-human β_2_-microglobulin (anti-β 2M). Following antibody treatments, cells were incubated with normal human serum as a source of complement. Normal human serum (NHS) was obtained from venous blood donated with consent by healthy volunteers (blood was allowed to clot for one hour at room temperature and for two hours on ice-water, then centrifuged, aliquoted and stored at minus 80°C). Specified dilutions of NHS were made in sterile isotonic barbital buffered saline, BBS^++ ^(0.15 mM Ca^++ ^and 1.0 mM Mg^++^), pH 7.3 and placed in frozen storage. Aliquots were thawed in warm water bath for 30 minutes prior to use.

### Lactate dehydrogenase-release-assay to detect complement-mediated damage

Lactate dehydrogenase (LDH) is an intracellular enzyme released into the extracellular environment when a cell is damaged by lytic perforations induced in the cell membrane via complement activation. Triplicate LDH assays were performed with 48-well plates (Fischer Scientific) with each sample containing approximately 1 × 10^5 ^MCF7 or Bcap37 cells. Incubations with NHS were for 30 min at 37°C; then 0.4 mM PMSF was added to prevent degradation of LDH by endogenously released intracellular proteases. Subsequently, these cells were incubated with the human serum for 4 additional hours at 37°C to elicit a maximum cellular response to complement activation. Application of PMSF simultaneously blocks new complement activation, yet we have observed that with additional time the cells with damaged membranes expand [26] and release LDH. Background detection of LDH was obtained with 1 × 10^5 ^cells incubated without antibodies in the presence of NHS, and was subtracted from experimental values. The LDH-assay solution (Sigma Diagnostics) was prepared according to the standard commercial protocol provided by Sigma Diagnostics. After each treatment group was incubated with NHS and specified antibody combinations, 50 μl samples were taken from the supernatant and added to 1 ml of the LDH-assay solution, mixed thoroughly and read at 340 nm. Total (100%) lysis was obtained by treating the cells with 1% Triton-X100 detergent. The percentage of lysis was determined by dividing the experimental LDH activity by the total lysis LDH value. At the conclusion of each LDH assay, Trypan Blue exclusion was used to corroborate the percentage of cells with membrane damage (percent of living cells) as compared to the LDH assay. An ANOVA statistical analysis revealed no significant variation between the commercial LDH detection assay and the Trypan Blue exclusion, revealing the LDH detection method to be a reliable method in the determination of complement-mediated cell membrane damage. In addition, Trypan Blue exclusion was used to verify viability of all cells at harvesting following control and experimental treatments.

### Flow cytometry: determination of cell cycle distribution and mCRP expression

Unsynchronized or glutamine synchronized treatment groups were prepared for flow cytometry analysis. Cells were harvested with cold Versene (Gibco) to obtain a single-suspension of cells and then centrifuged at 1000 rpm for 5 min at 27°C. Pellets were re-suspended in 1% bovine serum albumin (BSA) blocking solution and centrifuged at 1000 rpm (200 g) for 5 min at 27°C. Pellets were re-suspended in serum-free DMEM and aliquots of approximately 1 × 10^6 ^cells were probed with FITC-labeled monoclonal antibody to either CD55 (2 μg/ml mouse IgG1 BRIC-216, Serotec Inc.) or to CD59 (2 μg/ml mouse IgG2a, MEM43, Serotec Inc.). Isotype (negative) controls were non-specific FITC-labeled mouse immunoglobulins, IgG2a and IgG1. Concomitantly, cells were fixed with ice-cold 95% ethanol (pH 7.4). The cells were resuspended in 100 μl of 0.5 mg/ml propidium iodide and 0.1 mg/ml ribonuclease A in 0.15 M PBS. The treated cells were filtered through a 35-μm cell-strainer and analyzed using a FACSCalibur™ (Becton Dickinson) flow cytometer with a 488 nm argon-ion laser for excitation. Light emission as a function of propidium iodide intercalated within DNA (to identify the cell-cycle) was detected using a 585 nm bandpass filter and the data analyzed using ModFit LT™ (Verity) software. Populations of cells were identified in either G0-G1 or G2-M based on channel values dictated by the amount of propidium iodide present in each cell, representing different stages of cell cycle. All Diploid cells were identified, then separated according to channel values based on propidium iodide fluorescence at 585 nm. Cells identified as G0-G1 had channel values ranging approximately from 25 to 75. Cells identified as G2-M had channel values ranging approximately from 140 to 175. Gating was performed manually after identification of G0-1 and G2-M populations to further determine CD59 and CD55 Mean Fluorescent Intensity (MFI) among these distinct populations of cells at different stages in the cell cycle. The acquisition threshold was set at 5,000 cells using the ModFit LT software, which allowed 5,000 diploid cells to be identified in the G0-G1 and G2-M channel ranges, and then these cells were further evaluated for MFI of CD59 and CD55 expression. Using the negative background isotype controls (*non-specific FITC-labeled mouse immunoglobulins, IgG2a and IgG*), no binding of the negative control antibodies to the cell surface was identified among MCF7 and Bcap37 cells. The positive fluorescent antibody control (*fluorescently tagged anti-β*_2_*-microglobulin*) revealed uniform and near-ubiquitous expression of β_2_-microglobulin among all MCF7 and Bcap37 cells irrespective of cell cycle phase or glutamine synchronization. Data was analyzed using CellQuest™ (Becton Dickinson) software. Instrument performance was routinely monitored using DNA QC Particles and Calibrite™ beads (Becton Dickinson).

## Abbreviations

MCF7, adenocarcinoma breast cancer cell line; Bcap37, medullary breast cancer cell line; mCRPs, membrane complement regulatory proteins; CD55, Decay Accelerating Factor; CD59, protectin; LDH, lactate dehydrogenase; β2M, β_2_-microglobulin; FBS, fetal bovine serum.

## Competing interests

The author(s) declare that they have no competing interests.

## Authors' contributions

BSE carried out the experiments. MKBZ supplied the interpretation of the results and with RJB wrote the manuscript. All authors read and approved the manuscript.
